# The Skeleton

**Published:** 1864-01

**Authors:** 


### THE SKELETON.

Some few years ago, the London *Morning Chronicle* published a poem, entitled "Lines on a Skeleton," which excited much attention. Every effort, even to the offering a reward of fifty guineas, was vainly made to discover the author. All that ever transpired was, that the poem, in a fair, clerkly hand, was found near a skeleton of remarkable symmetry of form in the Museum of the Royal College of Surgeons, Lincoln's Inn, London, and that the Curator of the Museum had sent them to the *Morning Chronicle.*

### LINES ON A SKELETON.

Behold this ruin! 'Twas a skull,
Once of ethereal spirit full,
This narrow cell was Life's retreat,
This space was Thought's mysterious seat.
What beauteous visions filled this spot!
What dreams of pleasure long forgot!
Nor Hope, nor Love, nor Joy, nor Fear,
Has left one trace of record here.

Beneath this moldering canopy
Once shone the bright and busy eye:
But stare not at the dismal void.
If social Love that eye employed;
If with no lawless fire it gleamed,
But through the dews of kindness beamed,
That eye shall be forever bright
When stars and suns are sunk in night.

Within this hollow cavern hung
The ready, swift, and tuneful tongue.
If falsehood's honey it disdained,
And where it could not praise, was chained;
If bold in Virtue's cause it spoke,
Yet gentle Concord never broke—
This silent tongue shall plead for thee
When Time unvails Eternity.

Say, did these fingers delve the mine?
Or with its envied rubies shine?
To hew the rock, or wear the gem,
Can little now avail to them.
But if the page of Truth they sought,
Or comfort to the mourner brought,
These hands a richer meed shall claim
Than all that waits on Wealth or Fame.

Avails it, whether bare or shod,
These feet the path of Duty trod?
If from the bowers of Ease they fled,
To seek Affliction's humble shed.
If grandeur's guilty bribe they spurned,
And home to Virtue's cot returned
These feet with Angel's wings shall vie,
And tread the palace of the sky.

